# Higher blood nicotine concentrations following smokeless tobacco (pituri) and cigarette use linked to adverse pregnancy outcomes for Central Australian Aboriginal pregnancies

**DOI:** 10.1186/s12889-022-14609-4

**Published:** 2022-11-23

**Authors:** Angela Ratsch, Fiona Bogossian, Elizabeth A. Burmeister, BoMi Ryu, Kathryn J. Steadman

**Affiliations:** 1 Research Services, Wide Bay Hospital and Health Services, Nissen Street, Hervey Bay, QLD 4655 Australia; 2 University of the Sunshine Coast, Maroochydore, QLD 4558 Australia; 3 Department of Marine Life Science, Jeju National University, Jeju, 63243 Republic of Korea; 4 School of Pharmacy, The University of Queensland, Brisbane, QLD 4102 Australia

**Keywords:** Pregnancy, Maternal, Neonatal, Smoking, Smokeless tobacco, Pituri, Tobacco, Nicotine, Central Australia, Australian Aboriginal and Torres Strait Islander people

## Abstract

**Background:**

In central Australia, Aboriginal women use wild tobacco plants, *Nicotiana* spp. (locally known as *pituri*) as a chewed smokeless tobacco, with this use continuing throughout pregnancy and lactation. Our aim was to describe the biological concentrations of nicotine and metabolites in samples from mothers and neonates and examine the relationships between maternal self-reported tobacco use and maternal and neonatal outcomes.

**Methods:**

Central Australian Aboriginal mothers (and their neonates) who planned to birth at the Alice Springs Hospital (Northern Territory, Australia) provided biological samples: maternal blood, arterial and venous cord blood, amniotic fluid, maternal and neonatal urine, and breast milk. These were analysed for concentrations of nicotine and five metabolites.

**Results:**

A sample of 73 women were enrolled who self-reported: no-tobacco use (*n* = 31), tobacco chewing (*n* = 19), or smoking (*n* = 23). Not all biological samples were obtained from all mothers and neonates. In those where samples were available, higher total concentrations of nicotine and metabolites were found in the maternal plasma, urine, breast milk, cord bloods and Day 1 neonatal urine of chewers compared with smokers and no-tobacco users. Tobacco-exposed mothers (chewers and smokers) with elevated blood glucose had higher nicotine and metabolite concentrations than tobacco-exposed mothers without elevated glucose, and this was associated with increased neonatal birthweight. Neonates exposed to higher maternal nicotine levels were more likely to be admitted to Special Care Nursery. By Day 3, urinary concentrations in tobacco-exposed neonates had reduced from Day 1, although these remained higher than concentrations from neonates in the no-tobacco group.

**Conclusions:**

This research provides the first evidence that maternal pituri chewing results in high nicotine concentrations in a wide range of maternal and neonatal biological samples and that exposure may be associated with adverse maternal and neonatal outcomes. Screening for the use of all tobacco and nicotine products during pregnancy rather than focusing solely on smoking would provide a more comprehensive assessment and contribute to a more accurate determination of tobacco and nicotine exposure. This knowledge will better inform maternal and foetal care, direct attention to targeted cessation strategies and ultimately improve long-term clinical outcomes, not only in this vulnerable population, but also for the wider population.

**Note to readers:**

In this research, the central Australian Aboriginal women chose the term ‘Aboriginal’ to refer to themselves, and ‘Indigenous’ to refer to the broader group of Australian First Peoples. That choice has been maintained in the reporting of the research findings.

**Supplementary Information:**

The online version contains supplementary material available at 10.1186/s12889-022-14609-4.

## Background

In maternal non-Westernised populations, the use of smokeless tobacco (SLT), rather than smoking, is the normalised tobacco use behaviour [1]. Data from 140 countries shows that there are an estimated 90 million women > 15 years of age who use SLT products [1, 2]. The term SLT describes tobacco products that, rather than being burnt, are used as solids, pastes or powders in the nasal and oral cavities or on the skin [1]. Frequently used products include plug, snuff, toombak, paan, mishri, dissolvable tobacco lozenges and strips.

Nicotine is the principal pharmacologically active and addictive compound in tobacco. The use of SLT enables the extraction and absorption of nicotine and other compounds from the tobacco plant without exposure to the products of tobacco combustion associated with cigarette use [3]. In Australia, Aboriginal populations in the central regions use wild tobacco plants (*Nicotiana* spp.), known as *pituri,* as SLT [4]. The plants are dried, then combined with wood ash from specific trees, before being chewed into a mass (known as a quid) and retained behind the lip in the buccal space for extended durations. Analysis of dry leaf *Nicotiana* spp. preferred for use as pituri has demonstrated nicotine content as high as 11 mg/g [5]. The addition of the highly alkaline wood ash to the quid raises its pH [6], which increases the proportion of unprotonated (free-base) nicotine and enables increased nicotine absorption through the oral mucosa [7]. If the wet quid is removed from the lip, it is commonly placed behind the ear for later use, which creates a potential transdermal nicotine administration route [8]. The habitual use of pituri is established in early life and continues throughout pregnancy and lactation [9].

Nicotine is an agonist at nicotinic acetylcholine receptors (nAChRs) except at two nAChR subunits where it acts as an antagonist [3]. Nicotine produces wide-ranging generic, reproductive and pregnancy-specific biphasic responses, transitioning from initial simulation to depression in a dose-dependent and cumulative manner based on a range of individual factors including genetics, age, gender and pregnancy [3]. Nicotine transits across the placenta, becoming concentrated in the placenta and the foetus as demonstrated by higher ratios in the umbilical venous and arterial cord bloods than in maternal serum [10] and nAChR binding in the developing and immature foetus [11].

Nicotine has a half-life of about two hours and is metabolised via the CYP2A6 pathway primarily in the liver, with the brain, kidneys and lungs providing minor sites [12]. This short half-life produces large nicotine serum plasma fluctuations. Cotinine, the main nicotine metabolite [13], has a half-life of approximately 17 h and serum concentrations tenfold higher than nicotine, providing a more stable biomarker of nicotine exposure [14]. Other major metabolites include 3’-hydroxycotinine, nornicotine, nicotine glucuronide and nicotine-N-oxide [15]. Nicotine and its metabolites are excreted by the kidneys with the rate being dependent upon urinary pH. During pregnancy, there is a significant induction of CYP2A6 activity that increases nicotine plasma clearance by 60% and cotinine clearance by 140% resulting in an almost 50% reduction in cotinine half-life, down to 9 h from 17 h [16]. Clinically, these changes are important, as a reduction in nicotine and cotinine concentrations in late pregnancy compared with pre-pregnancy or early pregnancy may not necessarily reflect a decrease in nicotine exposure, but rather a more rapid metabolism [17]. The foetus and neonate have immature CYP2A6 activity, which decreases their ability to metabolise nicotine, resulting in longer plasma nicotine half-life relative to adults (11.2 h compared to 2 h), whereas cotinine elimination is similar to that of adults (16.3 h compared with 17 h) [18].

Extensive research over the past 60 years has demonstrated that maternal smoking, SLT use, and/or maternal exposure to second-hand cigarette smoke have adverse maternal and neonatal outcomes [19–23]. For the mother, these outcomes may include delayed conception, increased risk of miscarriage, ectopic pregnancy and elevated glucose [24–28]. At the foetal macro level, nicotine exposure is associated with reduced gestational length (preterm birth < 37 weeks gestation), low birthweight (LBW) and malformation [29]. Gestation length and birthweight are key markers of foetal health and are significantly influenced by the presence of elevated maternal glucose. Furthermore, the transition from a high glucose intrauterine environment to independent glucose control following birth can be challenging for neonates exposed to elevated maternal glucose [30–32] and increases the likelihood of neonatal admission to Special Care Nursery (SCN) [33, 34]. At the foetal micro level, nicotine has a teratogenic impact on neural nAChRs and neural physiology, increasing the risk for stillbirth, sudden infant death syndrome (SIDS) and Sudden Unexplained Death in Infancy (SUDI) for exposed offspring compared with non-exposed offspring [35–38].

Based on the evidence of nicotine exposure and adverse foetal impacts, nicotine is internationally classed as a Category D drug in pregnancy [39, 40]. The Australian Category D definition is “drugs which have caused, are suspected to have caused or may be expected to cause, an increased incidence of human foetal malformations or irreversible damage” [41]. The immediate adverse foetal and neonatal outcomes (i.e., pre-term birth, lower birthweight, stillbirth, SIDS) tend to overshadow and draw attention away from the adverse impact of in-utero nicotine exposure on foetal programming and the consequences to the offspring of lifelong increased risks for acute and chronic illness and disease including childhood obesity, early onset cardiovascular and respiratory disease and cognitive and behavioural barriers (attention-deficit/hyperactivity disorder (ADHD) and learning challenges) [42–46].

In Australia, maternal, pregnancy, birthing and neonatal information is gathered as part of the National Perinatal Data Collection [47]. For that collection, only maternal self-reported cigarette use is captured and reported twice; once before 20 weeks gestation, and once after 20 weeks gestation. That data shows the smoking rate for Indigenous mothers is 44% compared with 12% for non-Indigenous mothers [48]. The self-reported smoking rate for Northern Territory (NT) Indigenous mothers in the first 20 weeks of pregnancy is 52%, with a much lower rate of 30% reported for Indigenous women in the Alice Springs rural district [49]. This difference across the NT is surmised to be related to the traditional practice of chewing of tobacco (pituri) in the central region compared with the use of cigarettes in the north, commonly referred to as the Top End [50]. Maternal smoking and pregnancy outcomes from the Australian Mothers and Babies Report [51] and NT Mothers and Babies Report [52] identify that live-born babies born to Indigenous mothers who smoked were 18% more likely to be born pre-term compared with 12% of those born to non-Indigenous mothers who smoked. The data indicates that Indigenous maternal smoking accounts for 47% of low-birthweight babies compared to 12% for non-Indigenous babies [53].

Clinical findings from the cohort of 73 Central Australian Aboriginal mother-neonate pairs enrolled in this research [54, 55] indicate that the most clinically and statistically significant maternal outcomes were differences in hypertension and anaemia across the groups, and a higher rate of elevated maternal glucose in pituri chewing mothers (48%) compared with smoking mothers (22%) and no-tobacco using mothers (16%). The presence of elevated maternal glucose was associated with the birthweights of neonates, although the cohort neonate birthweight mean (3348 g) showed no difference between the nicotine user (or not) groups. When stratified for elevated maternal glucose, the neonates born to pituri chewers had the lowest mean birthweight (2906 g) compared to the neonates of the no-tobacco group (3242 g) and smokers (3398 g). Additionally, the neonates of chewers had lower APGAR scores and higher admission rates to SCN compared with the neonates of smokers (44% v 23%). Furthermore, there were differences in gender, and placental weight and diameter between the three exposure groups. The research question addressed in this paper is ‘what are the biochemical concentrations of nicotine and metabolites (NM) in a range of maternal and neonatal biological samples from mothers with differing levels of self-reported tobacco use?’. The aim is to describe the NM concentrations from maternal blood, maternal and neonatal urine, breast milk and amniotic fluid and examine any relationship with self-reported maternal tobacco exposure and maternal and neonatal outcomes.

## Methods

### Sampling frame, sample size, inclusion and exclusion criteria

The protocol for the research has been published elsewhere [56]. Briefly, all pregnant Central Australian Aboriginal women, ≥ 18 years of age, with a singleton pregnancy, ≥ 28 weeks gestation, and who planned to birth at the Alice Springs Hospital, NT, Australia were conveniently offered enrolment in the research. Based on the observed rates of pituri use (33%), smoking (33%) and no-tobacco use (33%), and the sample size from a similar project with Alaskan Native women [57], we expected that 20 mother-neonate pairs in each tobacco exposure group would enable statistical assessment of a difference between the biochemical concentrations of NM between the tobacco exposed groups (smoking and pituri use) and the no-tobacco exposed group, and provide data to inform the development of further research questions.

The health of Central Australian Aboriginal mothers is challenged by a range of circumstances and conditions which are known predictors for adverse pregnancy and neonatal outcomes, including low levels of education and access to antenatal care, high levels of unemployment, poor housing and nutrition, high rates of urinary tract and sexually transmitted infections, anaemia, diabetes and renal disease [58–60]. Given the endemic occurrence of these in the population, these were not used as exclusion criteria for eligibility. The only maternal exclusion criterion was self-reported dual pituri and cigarette use; excluded due to the lack of research resources to enable the distinction between nicotine biochemical concentrations absorbed through the maternal respiratory tract vs. through the maternal oral and transdermal routes [61].

### Participant characteristics, tobacco use and biological data collection tools

Three data collection strategies were used to address the research question, with the data being collected by Aboriginal Health Workers, Aboriginal Liaison Officers and midwives. The first data collection followed participant enrolment and comprised a semi-structured demographic and tobacco-use interview. The second strategy was the extraction of the pregnancy, labour and birth information from CARESYS® (the NT electronic medical record system). Any CARESYS® report of diabetes (pre-gestational or gestational) was categorized as “elevated glucose” and likewise any report of hypertension (pre-gestational or gestational) as “elevated blood pressure” for the analysis in this research. The third strategy was the collection of biological samples from the mother, placenta and neonate to measure the maternal, foetal and neonatal exposure and excretion of NM (Table 1).

**Table 1:** Biological samples collected for nicotine and metabolite (NM) measurement and their rationale for collection in response to the research question

Biological sample	Rationale for collection
Maternal venous blood	- indicates recency of maternal nicotine exposure [62]
Umbilical vein cord blood	- indicates nicotine placental transfer (and foetal exposure) from the maternal circulatory system
Umbilical artery cord blood	-indicates foetal nicotine excretion via the circulatory system (and return to the mother)
Amniotic fluid	- demonstrates foetal nicotine exposure directly through the amniotic membrane, and foetal nicotine excretion via the foetal kidneys and lungs [11, 63–65]
Maternal urine	- indicates maternal nicotine excretion via urinary system [3]
Neonatal urine	- comparison of Day 1 and Day 3 urine with umbilical vein and umbilical artery cord bloods may demonstrate neonatal nicotine absorption, metabolism and excretion [18]
Colostrum and/or breast milk	- indicates nicotine excretion and possible route of post-birth nicotine exposure [66, 67]

### Biological data collection, storage and analysis

Biological samples were collected as they became available and when sampling did not impact clinical care. Maternal plasma and non-sterile urine were concurrently collected with other plasma and urine collections to minimise participant discomfort. A sample of amniotic fluid that was not visibly contaminated with maternal blood or meconium was obtained at caesarean section (CS). Cord bloods were collected as per standard umbilical cord blood collection procedures following the complete expulsion of the placenta from the uterus and the complete separation of the placenta from the neonate. Neonatal urine collection bags were placed on the neonate after birth and again on Day 3, and urine was collected when available and if visibly uncontaminated with meconium. Mothers were encouraged to collect their breast milk when that became available. Under the counsel of the Aboriginal advisory group, no biological samples were collected from the placentas or neonates who were stillborn.

Participant samples were frozen (-80 °C) until their transport for analysis. The samples were batch analysed for nicotine, cotinine, 3’-OH-cotinine, nornicotine, nicotine-N-oxide and nicotine glucuronide using LC–MS/MS following the methodology contained in Additional file 1. The analysis was conducted by one scientist (BM) who was blinded to the self-reported tobacco use status of participants.

### Data analysis

Maternal self-reported tobacco use at initial enrolment interview was used to categorise participants into one of the three tobacco-use groups: (a) no-tobacco user, (b) chewer, or (c) smoker. The concentration (ng/mL) of nicotine, cotinine, 3’-hydroxycotinine, nornicotine, nicotine-N-oxide and nicotine glucuronide in each sample were transformed to nmol/mL and summed to give a total nicotine and metabolite (NM) molar concentration for data analysis [68]. Analytes below the limit of detection (LOD) were recorded as the LOD divided by the square root of two [69]. NM concentrations were compared with maternal, birthing and neonatal variables of clinical interest; elevated maternal glucose, hypertension, anaemia, placenta characteristics, and neonatal gender, birthweight, gestational age, APGAR score and admission to SCN [54, 55].

Data from the interview, CARESYS® files and biochemical results were de-identified and imported into SPSS® (IBM® SPSS_®_ Statistics 20) and Stata 17 (StataCorp, Texas) for the analyses. Descriptive statistics including frequencies, percentages, means and 95% confidence intervals (CI) were calculated and reported. Box and whisker graphs were used to summarise median, interquartile ranges and outlier data according to tobacco-use groups. Where individual outcomes conflicted with the participant’s self-reported tobacco use status, these were retained and included in the analysis of the self-reported tobacco-use group.

### Ethical considerations

The research design and methodology were informed and overseen by a regional Aboriginal Women’s Council. Ethical approval was obtained from the Central Australian (#2010.06.04) and The University of Queensland Human Research Ethics Committees (#2,010,000,548 and #2,015,001,429). All participants provided written informed consent prior to enrolling in the research and all methods were performed in accordance with the guidelines and regulations of the National Statement for the Ethical Conduct in Human Research [70].

## Results

### Nicotine and metabolite concentrations

A total of 73 mothers and 73 neonates participated in the research. Two neonates were stillborn at 40 weeks gestation; their mother’s pregnancy and their birth data are reported, but no biological samples were collected from these neonates. For the other 144 participants, it was not possible to collect or analyse all samples for every participant because the sample was either declined by the participant, or was required for clinical care, or was contaminated with other biological fluid, or was inadequately labelled, or there was an inadequate sample volume for analysis. Additionally, six mothers birthed at other hospitals or before arrival at Alice Springs Hospital. The following were analysed: 61 maternal blood, 37 cord blood, 6 amniotic fluid, 59 maternal urine, 32 neonatal urine Day 1, 20 neonatal urine Day 3, and 39 breast milk samples. For the umbilical cord blood collections, almost half (11/37) were insufficiently labelled to clearly identify the sample by vessel of origin (artery or vein). Therefore, the unidentified cord blood results have been included in the total cord blood results in this report, with the mean value of the unlabelled samples and labelled samples reported if more than one sample was available for each participant.

**Fig. 1:**
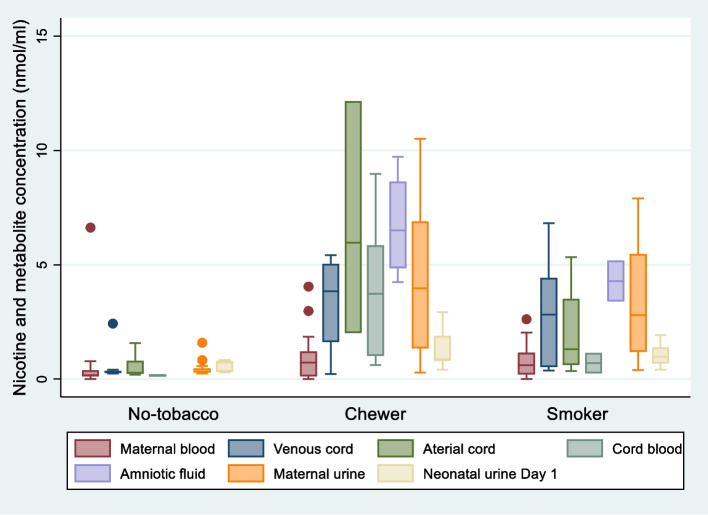
Total nicotine and metabolite concentrations (nmol/mL) in biological samples. Boxplot indicating the median, interquartile ranges, upper and lower adjacent values and outliers of total nicotine and metabolite concentrations measured in biological fluids from mothers and their neonates. Mothers (*n* = 73) self-reported their tobacco use as no-tobacco, chewing or smoking

Missing data assessment revealed only the mother’s samples had individual analyte data below the LOD: mothers breast milk samples (4%—72 individual analytes) and mothers blood samples (35 analytes—2% of total measured) (Table 2). All samples contained cotinine levels above the LOD. Cotinine concentrations (ng/mL) in maternal blood and urine form the basis of most maternal tobacco exposure reports [68] and in this paper, the cotinine concentrations from each biological sample together with the summed NM molar concentrations (nmol/mL) are reported in Table 2. The no-tobacco group provided the control for this research showing mean maternal blood cotinine concentrations of 42 ng/mL and NM concentrations of 0.44 nmol/mL plus mean maternal urine cotinine concentrations of 13 ng/mL and 0.43 nmol/mL with similar values in the cord blood and neonate urine (Table 2). This group included one outlier with an exceedingly high blood NM concentration (6.63 nmol/mL) indicating very recent tobacco use even though she self-identified as a no-tobacco user (Fig. 1).

**Table 2:** Cotinine (ng/mL) and total nicotine and metabolite concentration (NM, nmol/mL) mean and 95% confidence interval for maternal and neonatal biological fluids according to maternal self-reported tobacco use (*n* = 73)

	Self-reported tobacco use								
	No-tobacco *n* = 31			Chewer *n* = 19			Smoker *n* = 23		
	n	Cotinine, ng/Ml mean (95% CI)	Total NM, nmol/mL mean (95% CI)	n	Cotinine, ng/mL mean (95% CI)	Total NM, nmol/mL mean (95% CI)	n	Cotinine, ng/mL mean (95% CI)	Total NM, nmol/mL mean (95% CI)
Maternal blood, *n* = 61	28	42.06 (-23.42—107.54)	0.44 (0.13—0.87)^1^	15	121.57 (25.65—217.49)	0.90 (0.38—1.43)^2^	18	78.97 (30.47—127.46)	0.73 (0.43—1.03)^3^
Cord blood ^5^, *n* = 37	15	19.96 (-2.97—42.90)	0.40 (0.19—0.61)	10	510.29 (207.52—813.05)	4.07 (1.81—6.34)	12	306.37 (11.87—500.88)	2.39 (1.08—3.70)
Vein cord blood, *n* = 26	12	35.25 (-22.82—93.31)	0.48 (0.09—0.87)	4	484.76 (-77.63—1047.15)	3.33 (-0.34—7.00)	10	398.71 (127.43—670.01)	3.01 (1.25—4.77)
Artery cord blood, *n* = 18	7	10.05 (6.97—13.12)	0.52 (0.04—0.99)^4^	3	1050.50 (-1066.84 -3167.84)	6.71 (-6.00—19.42)	8	264.22 (34.33—494.12)	2.07 (0.52—3.61)
Unknown cord blood vessel, *n* = 11	3	9.97 (7.35—12.59)	0.17 (0.03—0.30)	6	425.51 (112.75–738.28)	3.98 (0.71—7.26)	2	77.00 (-780.76—934.77)	0.70 (-4.93—6.32)
Amniotic fluid, *n* = 6	0	-	-	4	478.65 (176.89—780.42)	6.74 (2.90—10.58)	2	368.84 (-999.25—1736.93)	4.29 (-7.05—15.62)
Maternal urine, *n* = 59	26	13.19 (11.86—14.52)	0.43 (0.32—0.55)	16	138.90 (63.69—214.11)	4.30 (2.53—6.07)	17	97.85 (39.01—156.70)	3.30 (2.08—4.53)
Neonatal urine Day 1, *n* = 32	16	12.54 (11.96—13.12)	0.62 (0.50- 0.74)	8	26.58 (13.18—39.99)	1.30 (0.57—2.04)	8	26.54 (3.98—49.11)	1.05 (0.62—1.48)
Neonatal urine Day 3, *n* = 20	13	12.30 (11.42—13.18)	0.48 (0.37—0.58)	4	28.93 (-2.00–59.86)	0.69 (0.53—0.86)	3	44.63 (-67.37—156.63)	0.90 (0.50—1.30)
Breast milk, *n* = 39^6^	19	3.05 (-1.31—7.41)	0.06 (0.02—0.10)	10	28.65 (3.33—53.97)	0.31 (0.10—0.52)	10	5.46 (1.10—9.82)	0.06 (0.02—0.11)

Analyte levels below level of detection

^1^Nicotine *n* = 3, Nornicotine *n* = 9, Nicotine-N-oxide *n* = 8

^2^Nornicotine *n* = 6; Nicotine-N-oxide *n* = 3

^3^Nornicotine *n* = 6

^4^Nicotine-N-oxide *n* = 3

^5^Cord blood value is the mean value of all cord blood samples per participant. 18 cord blood samples from 11 participants were not labelled with source vessel (i.e., arterial or venous). If more than 1 sample from each participant, then mean value of samples reported

^6^Breast milk: Nicotine *n* = 18, Nornicotine *n* = 17, Nicotine glucuronide *n* = 36, Nicotine-N-oxide *n* = 3

The maternal cotinine and NM concentration range between individuals in both the chewer and smoker groups varied widely (Table 2 and Fig. 1). Even with this wide range, differences between the no-tobacco and the chewers and smoker groups are evident. As expected, tobacco smokers and chewers had considerably higher mean cotinine and NM values than the no-tobacco group concentrations (Table 2). Maternal blood cotinine concentrations were almost double for smokers (79 ng/mL) and triple for chewers (121 ng/mL) compared to the no-tobacco result of 42 ng/ml, and maternal urine NM concentrations were approximately 10 times greater (3.3 nmol/mL smokers and 4.3 nmol/mL for chewers compared to 0.40 nmol/mL for no-tobacco users). The chewer group displayed the highest mean NM concentrations in almost all of the biological fluids reported (all except the neonatal Day 3 urine). The mean NM value for the amniotic fluid from chewers was the highest of all biological fluids (6.74 nmol/mL), followed by their arterial cord blood (6.71 nmol/mL), and maternal urine (4.30 nmol/mL).

Across the groups, for mothers with both blood and urine available (*n* = 55) the mean NM maternal urine (2.42 nmol/mL, 95% CI 1.67–3.18) far exceeded the mean NM maternal blood concentration (0.80 nmol/mL, 95% CI 0.49–1.10).

Transfer of NM between the maternal and foetal circulation at the time of birthing is reported by umbilical cord blood. Notably, NM concentration in cord blood was more than triple maternal blood for the smokers (2.39 nmol/mL vs 0.73 nmol/mL), and more than four times for the chewers (4.07 nmol/mL vs 0.90 nmol/mL). For the 32 participants for whom both cord blood and maternal blood samples were analysed, NM concentrations were higher in cord blood than in their maternal blood (mean cord blood 2.21 nmol/mL, 95% CI 1.27 – 3.14; maternal blood 0.79 nmol/mL, 95% CI 0.35 – 1.23).

Amniotic fluid is a further indicator of foetal exposure to nicotine. Amniotic fluid uncontaminated with maternal blood or meconium was very difficult to acquire at the time of vaginal birth. The samples that were obtained (n = 6) were from caesarean section births. Consequently, with so few samples it is not possible to make comparisons, however, the six tobacco users’ mean NM concentration in amniotic fluid was significantly higher than their maternal blood NM concentrations (amniotic fluid 5.92 nmol/mL, 95% CI 3.48 – 8.37; maternal blood 1.35 nmol/mL, 95% CI 0.37—2.32).

Neonatal excretion of NM was measured in neonatal urine at Day 1 and Day 3 with the chewers and smokers having higher Day 1 mean concentrations than the no-tobacco group (1.30 nmol/mL, 95% CI 0.57—2.04; 1.05 nmol/mL 95% CI 0.62—1.48; 0.62 nmol/mL 95% CI 0.50—0.74 respectively). Day 3 results show declines in each group (chewers 0.69 nmol/mL, 95% CI 0.53—0.86; smokers 0.90 nmol/mL 95% CI 0.50—1.30; no-tobacco group 0.48 nmol/mL 95% CI 0.37—0.58). Neonates from the three groups with both Day 1 and Day 3 results (*n* = 15) showed a decline in mean NM concentration over that period of time (Day 1 neonatal urine 0.87 nmol/mL, 95% CI 0.49—1.26, Day 3 neonatal urine 0.56 nmol/mL, 95% CI 0.42—0.69,). From neonates with both urine and cord blood samples, the mean NM urine levels were lower than cord blood concentrations (*n* = 17; Day 1 neonatal urine 1.09 nmol/mL, 95% CI 0.71 – 1.46, all cord blood 2.66 nmol/mL, 95% CI 1.09 – 4.23).

The continued exposure of neonates to nicotine via breastfeeding was also considered (Table 2). Concentrations were lower in the breast milk than all other biological fluids, however the highest mean NM concentrations were recorded in chewers (0.31 nmol/mL, 95% CI 0.10—0.52) followed by the smokers (0.06 nmol/mL, 95% CI 0.02—0.11) and non-tobacco users (0.06 nmol/mL, 95% CI 0.02—0.10).

### Relationship of nicotine concentrations with maternal outcomes

In the cohort of 61 mothers with maternal blood collections, 33% of tobacco users had elevated maternal glucose (47% of chewers and 22% of smokers) compared to 18% of the no-tobacco group (Table 3). Mean maternal blood NM for chewers with elevated blood glucose was almost double chewers without elevated glucose (1.57 nmol/mL compared with 0.77 nmol/mL). Similarly, maternal blood NM levels for smokers with elevated blood glucose (1.37 nmol/mL) were higher for than smokers without elevated glucose (0.80 nmol/mL).

**Table 3:** Maternal blood total nicotine and metabolite concentration (NM) according to maternal self-reported tobacco use (*n* = 61), and subdivided according to maternal, neonatal and placental characteristics, outcomes and complications. Data are the frequency, mean and 95% confidence interval for NM in nmol/ml. Birthweight (g), placenta weight (g) and placenta size (cm x cm) are shown in italics

	No-tobacco			Chewer			Smoker		
	n	mean	95% CI	n	mean	95% CI	n	mean	95% CI
**Maternal outcomes (*n* = 61)**									
Maternal blood NM, nmol/mL	28	0.48	0.01 – 0.96	15	1.14	0.53 – 1.75	18	0.93	0.60 – 1.26
Elevated glucose									
Yes *n* = 16	5	0.18	0.14 – 0.21	7	1.57	0.22 –- 2.93	4	1.37	0.02 – 2.73
No n = 45	23	0.55	-0.02 – 1.13	8	0.77	0.36 – 1.18	14	0.80	0.48 – 1.13
Hypertension									
Yes *n* = 12	7	1.12	-1.12 – 3.37	1	1.54	-	4	0.56	0.11 – 1.01
No *n* = 49	21	0.27	0.19 – 0.36	14	1.11	0.46 – 1.77	14	1.03	0.63 – 1.44
Anaemia									
Yes *n* = 13	8	0.33	0.14 – 0.52	1	0.72	-	4	0.74	-0.65 – 2.14
No *n* = 48	20	0.55	-0.12 – 1.22	14	1.17	0.52 – 1.81	14	0.98	0.63 – 1.33
**Neonatal outcomes (*n* = 61)**									
* Mean birthweight, g*	*28*	*3279*	*3051* – *3506*	*15*	*3253*	*2834* – *3673*	*18*	*3338*	*2828* – *3847*
Gestation									
< 37 weeks *n* = 7	3	2.45	-6.54 – 11.44	2	1.86	-12.40 – 16.11	2	0.70	-5.04 – 6.43
≥ 37 weeks *n* = 54	25	0.25	0.175 – 0.321	13	1.03	0.40 – 1.67	16	0.96	0.60 – 1.32
Exposure to elevated glucose									
Yes *n* = 16	5	0.18	0.14 – 0.21	7	1.57	0.21 – 2.93	4	1.371	0.01 – 2.73
* mean birthweight, g*	*5*	*3466*	*2223* – *4709*	*7*	*3794*	*3287* – *4301*	*4*	*3127*	*734* – *5520*
No *n* = 45	23	0.55	-0.03 – 1.13	8	0.77	0.36 – 1.18	14	0.80	0.48 – 1.13
* mean birthweight, g*	*23*	*3238*	*3030* –*- 3445*	*8*	*2780*	*2293* – *3267*	*14*	*3398*	*2871* – *3925*
Gender									
Male *n* = 32	16	0.69	-0.16 – 1.54	9	1.40	0.36 – 2.44	7	0.77	0.28 – 1.27
* mean birthweight, g*	*16*	*3209*	*2931* – *3486*	*9*	*3457*	*2920* – *3995*	*7*	*3723*	*2857* – *4589*
Females *n* = 29	12	0.21	0.13 – 0.29	6	0.76	0.32 – 1.20	11	1.03	0.54 – 1.52
* mean birthweight, g*	*12*	*3372*	*2941* – *3802*	*6*	*2947*	*2111* – *3783*	*11*	*3093*	*2392* – *3793*
Neonate admitted to SCN^1^									
Yes *n* = 22	11	0.84	-0.45 – 2.13	7	1.81	0.61 – 3.01	4	1.17	-0.46 – 2.81
No *n* = 39	17	0.25	0.15 – 0.35	8	0.56	0.22 – 0.90	14	0.86	0.55 – 1.17
APGAR at 5 minutes^1^									
< 7 *n* = 6	3	0.22	-0.12 – 0.56	2	2.40	-18.57 – 23.36	1	1.04	-
≥ 7 *n* = 53	24	0.53	-0.02 – 1.08	13	0.95	0.46 – 1.44	16	0.91	0.54 – 1.28
**Placental characteristics**^1^ **(*n* = 40)**									
Maternal blood NM, nmol/mL	21	0.55	-0.09 – 1.19	10	0.99	0.35 – 1.63	9	0.76	0.45 – 1.08
* Placenta size,* cm x cm	*21*	*299*	*270* – *327*	*10*	*280*	*213* – *347*	*9*	*250*	*198* – *302*
* Placenta weight,* g	*21*	*572*	*524* – *620*	*10*	*488*	*376* – *599*	*9*	*667*	*268* – *1065*

^1^Data for neonatal live births only (*n* = 59)

Participants with anaemia tended to have lower NM concentrations (chewers 0.72 nmol/mL, smoker 0.74 nmol/mL, no-tobacco users 0.33 nmol/mL) compared with those without anaemia (chewers 1.17 nmol/mL, smokers 0.98 nmol/mL, no-tobacco users 0.55 nmol/mL), though the number of participants with anaemia was low and variability was high (Table 3). The relationship between maternal hypertension and NM concentrations was inconsistent between groups (Table 3). Smokers with hypertension had a lower mean NM (0.56 nmol/mL) than smokers without hypertension (1.03 nmol/mL) while the no-tobacco hypertensive group mean NM (1.11 nmol/mL) was higher than those no-tobacco users without hypertension (0.27 nmol/mL), although when data from one very high NM level (6.63 nmol/mL) in the no-tobacco group was omitted from analysis, the mean NM for the no-tobacco hypertensive group was 0.20 nmol/mL. The single chewer with hypertension had a high maternal blood NM (1.54 nmol/mL) compared to the mean for chewers without hypertension (1.11 nmol/mL).

### Relationship between maternal NM levels with neonatal and placental outcomes

Between groups, there were fewer males born in the smoking group compared with the chewing group and the no-tobacco group (43%, 57% and 60% respectively). When considered by NM concentrations and neonatal gender, maternal blood NM levels were higher in male neonates (0.91 nmol/mL) compared to females (0.63 nmol/mL). The maternal NM levels were higher in both the chewing group and no-tobacco group for male neonates (1.40 nmol/mL and 0.69 nmol/mL) compared with female neonates (0.76 nmol/mL and 0.21 nmol/mL) and the reversal was seen in neonates of smoking mothers (males 0.77 nmol/mL and female 1.03 nmol/mL).

The average birthweight for the 61 neonates with maternal blood available was 3290 g, with little difference between the three tobacco exposure groups (Table 3). Results from both chewers and smokers demonstrated the mean birthweight for male neonates was 510 and 630 g heavier than the female neonates of chewers and smokers respectively, but in the no-tobacco group males and females were very similar weights (3209 g and 3372 g respectively).

In the chewers, neonates exposed to maternal elevated glucose were also exposed to higher maternal NM (1.57 nmol/mL) and weighed 1014 g more than the neonates of chewers who were not exposed to elevated glucose and were exposed to lower maternal NM (0.77 nmol/mL). This same trend existed for smokers only when a very small (900 g) pre-term 27-week gestation neonate was excluded from the elevated glucose group for analysis, in which case neonates from smokers exposed to elevated glucose weighed 472 g more than those not exposed (3869 g versus 3398 g).

SCN admission was more frequent for neonates of chewers (47%) than smokers (22%) and no-tobacco-users (39%), and those admitted had higher maternal blood mean NM concentrations than those not admitted (chewers 1.81 nmol/mL, smokers 1.17 nmol/mL, no-tobacco 0.84 nmol/mL, vs. chewers 0.56 nmol/mL; smokers 0.86 nmol/mL, no-tobacco 0.25 nmol/mL). Very few neonates had APGAR scores of < 7 at five minutes (Table 3), but for the chewers, those with scores < 7 had higher maternal blood NM concentrations than those with ≥ 7 scores (2.40 nmol/mL versus 0.95 nmol/mL) in comparison with the no-tobacco users and the smokers. Of the 61 births, 40 placentas were able to be examined (Table 3). There was a trend towards higher NM concentrations associated with smaller and lighter placentas. In the smoking group there was one extremely large placenta (weight was verified, 1960 g), that if omitted, brought the average placenta size and weight to 259 cm^2^ and 505 g.

## Discussion

This paper reports the concentration of NM in a wide range of biological samples from prospectively and conveniently enrolled Central Australian Aboriginal pregnant women at hospital for the birth of their baby; mothers self-reported their tobacco status as being a chewer, a smoker, or a no-tobacco user. We also considered NM concentrations in relation to the maternal, birthing, placental and neonatal characteristics and outcomes previously described for these families within the overarching research [54, 55].

The population consisted of 58% self-reported tobacco users (SLT 26%, smokers 32%), however in the self-reported no-tobacco group, several high maternal cotinine and NM concentrations were indicative of tobacco exposure. Re-categorisation of participants based on cotinine or NM concentrations would have enabled a more robust statistical analysis including correlation and regression, however this re-categorisation was not conducted for the following reasons. Firstly, urinary cotinine levels (i.e., nicotine metabolism) is commonly used as a cut-off value to indicate tobacco use in pregnancy, yet the literature reports a wide range in cut-off values for example 250 ng/mL [71], 82 ng/mL [72], and 42 ng/mL [73]. Secondly, nicotine metabolism is highly individual and is influenced by previous exposure, recency of exposure prior to collection, tobacco formulation, diet and body weight. Importantly, evidence shows that following equivalent tobacco exposure, ethnically different populations have varied nicotine intake and cotinine clearance [74, 75] and specific genomic variations have been identified and contribute to the biological variation in NM findings in different populations [76]. As yet, genome research related to the metabolism of nicotine in pregnant Australian Aboriginal populations has not been conducted. Furthermore, there were dissimilarities in study designs and methods between this research and other studies including differences in laboratory techniques and analysis equipment. Adding further complexity to the comparison of study findings, is the global diversity of products described as SLT, and their corresponding different curing, production, admixtures, methods of administration, and amounts and frequency of use. Given these issues it was considered premature to extensively compare nicotine absorption and clearance concentrations, and maternal and neonatal outcomes in this research cohort with wider findings. Lastly, the Australian maternity data collection is based on self-report, and by retaining the self-reported categorisation it is possible to compare the research findings with the National Perinatal Data Collection.

### Maternal nicotine exposure and outcomes

In Australia, the National Perinatal Data Collection reports on mothers’ use of cigarettes twice during pregnancy and there is no opportunity to report SLT or nicotine containing products such as e-cigarettes, nicotine gum, patches, mists, tooth powder or lozenges. Accordingly, the pituri users’ outcomes in this research will have been recorded in the National Perinatal Data Collection as “no-tobacco use”, thus, incorrectly reporting the nicotine exposure outcomes for Aboriginal and Torres Strait Islander mothers and babies at the local, Territory, national and international level. Modifying the pregnancy health assessment and data collection tools to record the broader range and use of nicotine containing products will enable a more inclusive and discriminative assessment of their effects on contemporary Australian pregnancies and outcomes.

The National Perinatal Data Collection reports the rate of smoking in pregnancy by Indigenous women across Australia is 44% [48], and the reported rate of smoking in the first 20 weeks of gestation is 52% across the NT. The reported rate of smoking for Indigenous women in the Alice Springs area is 30% [49] and comparable to the finding in this research of 32%. In this research, which captured SLT use as well as cigarette use, there were indications of higher mean concentrations of NM in the chewer group’s maternal blood, umbilical cord blood, amniotic fluid, neonatal urine Day 1 and breast milk in comparison with the smoker and no-tobacco use groups. Higher concentrations of NM in both tobacco user groups were aligned with several clinically important maternal differences, specifically in the rate of elevated glucose, hypertension and anaemia.

In general populations, smoking has been identified as a risk factor for the development of diabetes [77, 78], and while the development of impaired glucose regulation is a normal physiological change in pregnancy [79], both pre-pregnancy and maternal smoking increase the risk of gestational diabetes [26–28]. Globally, the rate of diabetes in pregnancy is higher in Indigenous populations [80] and there is also a higher use of tobacco in Indigenous populations [1]. At the Australian level, the incidence of gestation diabetes has increased from 5.2% in 2000–2001, to 9.3% in 2012–2013, to 15.1% in 2016–2017 [81]. In this research, the high maternal blood NM levels in the tobacco-exposed groups with elevated glucose, and the lower NM levels in the tobacco-exposed groups without elevated glucose suggests the involvement of nicotine in insulin resistance or glucose metabolism as opposed to a mechanism related to only the combustion of tobacco. The presence of elevated glucose impacts the mother during pregnancy as well as her longer-term health, with approximately 50% of affected mothers going on to develop diabetes within 10 years of first diagnosis of gestational diabetes [82].

While nicotine is a potent vasoconstrictor [3], counterintuitively smoking in pregnancy results in a dose-dependent decrease in hypertension [20, 83–85]. Our data for smokers supports this assertion, with fewer cases of elevated hypertension in smokers than in no-tobacco users. There is evidence of hypertension in smoking participants with lower mean maternal blood NM concentrations compared with an absence of hypertension in those with higher NM levels. Chewing tobacco was expected to follow the same trend [31] (i.e., higher NM and an absence of hypertension) and this was evidenced by only one chewer having a record of hypertension; this participant had a high NM (1.54 nmol/mL). In the no-tobacco group, with the removal of the one high NM result (6.63 nmol/mL) from a participant with hypertension, the mean NM result was 0.20 nmol/mL for those with hypertension, and 0.27 nmol/mL in those without hypertension.

Maternal anaemia occurred less often in participants with higher mean NM concentrations compared with those with lower mean concentrations. These findings are contrary to the literature which shows that smoking and SLT use in pregnancy is associated with maternal anaemia [22, 86–88], however there may be SLT product differences in other populations, and endemic confounders in this population that explain this finding. In the geographical region, intestinal worms (*Hymenolepis nana*) are endemic, and anaemia is associated with approximately 18% of infections [89]. Nicotine exposure is a treatment for intestinal worms in humans and animals [90–92], and in this population, we hypothesise that exposure to nicotine from pituri chewing and smoking may decrease the worm burden in a dose-dependent manner and thereby decrease the resultant anaemia from this parasite.

### Neonatal nicotine exposure and outcomes

Nicotine readily transits from the maternal circulation to the placenta and much has been written around the neonatal outcomes of in-utero exposure during pregnancy, birth and early infancy [93], however longer-term outcomes are being demonstrated including that in-utero nicotine exposure permanently impacts the foetal pancreas and results in a loss of beta cell mass, leading to a life-long increased risk of impaired glucose and insulin homeostasis, childhood and adult obesegenesis [94–97], and childhood [98] and adult hypertension [99] and type 2 diabetes [100].

In this research, evidence of neonatal exposure to nicotine was demonstrated with NM concentrations in arterial and venous cord blood and amniotic fluid in those exposed to tobacco through smoking or chewing and notably, these concentrations were significantly higher than the maternal blood concentrations. The unlabelled cord blood values are comprised of a mixture of venous and arterial samples, so while the arterial cord blood NM concentrations may be higher than venous cord blood from chewers (Table 2), these are based on only three participants for whom both labelled samples were available, and the relationship is reversed for the eight smokers for whom there were both cord blood samples.

As observed in this research through NM concentrations measured in urine, the neonate and the mother are able to excrete nicotine via the kidney [18, 101] and Day 1 NM urine concentration is a useful biomarker for nicotine exposure. Following birth and the discontinuation of the supply of nicotine via the placenta, neonatal urine from chewers and smokers declined in NM concentration from Day 1 to Day 3. Whilst this finding provides some reassurance of the neonatal ability to excrete the NM resulting from in-utero exposure, the mean concentrations in both the neonates of chewers and smokers did not equate to that of the neonates from the no-tobacco use group by Day 3. However, we were not able to take into account hydration status with these small volumes, so no inferences can be made.

There is potential for continued neonatal nicotine exposure following birth through breast milk [102]. NM concentration in breast milk from smokers was low and similar to the no-tobacco group (0.064 nmol/mL and 0.061 nmol/mL respectively) while breast milk of chewers was relatively higher (0.309 nmol/mL). Pituri can be used discretely, and continuously throughout the hospital stay which may account for these higher levels. SLT may also be associated with slower nicotine clearance from breast milk in chewers than smokers, as breast milk is clear of nicotine in smokers after four hours of abstinence but is still present in snus (SLT) users’ after abstaining from tobacco use for 11 h [98].

We anticipated higher rates of SCN admission for the smoker group than the no-tobacco group [103], but the opposite was true, with fewer smoker’s compared to no-tobacco neonates treated in SCN. Pituri chewers were most likely to be admitted and this was associated with high mean maternal blood NM, indeed, all mothers whose neonates were admitted to SCN had higher blood NM than mothers of non-admitted neonates.

Literature from smoked tobacco research indicates a reduction in male newborns in the presence of maternal and/or paternal cigarette smoking in a nicotine dose-dependent manner [104–106], and our smokers produced 43% male births, down from the worldwide average of 51.4% [107]. There is emerging evidence that changes to DNA methylation as a result of maternal smoking are greater in male offspring than female offspring [108]. However, while smoking would be expected to be associated with a decrease in birthweight, particularly in male offspring [103], we found male neonates from smokers were 500 g heavier than those from the no-tobacco group, while females were on average 280 g lighter. The tobacco chewer group, despite exhibiting higher mean NM levels in their blood, had 60% male births and less impact on male birthweight.

Maternal smoking and SLT use increases the risk of a small for gestation age (SGA) neonate [109, 110]. Likewise, elevated maternal glucose increases the likelihood of an earlier (spontaneous or induced) birth, and increases the likelihood of an SGA or a large for gestation age (LGA) neonate dependent upon neonatal genome [111, 112]. In Australian Aboriginal pregnancies, the immediate foetal impact of exposure to pre-gestational and gestational diabetes is different to that evidenced in Australian non-Aboriginal pregnancies. In the presence of pre-gestational diabetes, there is a slightly higher incidence of an LGA birth in Aboriginal pregnancies compared with non-Aboriginal pregnancies (32.9% versus 32.7%) and an increase in SGA births (8.2% versus 4.6%). In the presence of gestational diabetes there is an increased incidence of an LGA neonate in Aboriginal pregnancies compared with non-Aboriginal pregnancies (21.1% versus 13.3%) and the reverse for the incidence of an SGA birth in Aboriginal pregnancies compared with non-Aboriginal pregnancies (7.1% versus 8.3%) [111, 112].

In this research, the medical record of participants often recorded both pre-gestational and gestational diabetes, and accordingly, this variable was dealt with as one variable. The research findings show that whilst the mean birthweight for each group across the cohort was similar, lower birthweights were seen in the presence of tobacco exposure when maternal elevated glucose was absent. Tobacco-exposed women with elevated blood glucose had higher maternal blood NM concentrations than their group counterparts without elevated glucose and had analogous higher birthweight neonates. In the chewers, neonates exposed to elevated glucose and higher maternal NM weighed 1014 g more than the neonates of chewers not exposed to elevated glucose and lower maternal NM. This same trend existed for smokers only if a very small (900 g) pre-term 27-week gestation neonate was excluded from the elevated glucose group for analysis, in which case neonates from smokers exposed to elevated glucose weighed 472 g more than those not exposed.

## Limitations

We grouped participants by their response to the interview question “have you smoked cigarettes or chewed tobacco in this pregnancy?” Surprisingly, participants with visible oral pituri quids answered “no” to this question. It became apparent that chewers did not consider pituri as tobacco. This finding was later confirmed in ethnobotanical interviews with senior Aboriginal women who rejected the notion that pituri was a tobacco plant [9]. The interview question was subsequently changed to include “have you chewed pituri or mingkulpa or tobacco in this pregnancy?” Nevertheless, early participant enrolments may have been incorrectly categorised, and this may explain some high NM readings in the no-tobacco use group.

Biological samples were taken at a single time point, so the NM values do not accurately reflect the totality of tobacco and nicotine exposure during the pregnancy, and instead indicate the extent of recent exposure. This is particularly relevant when comparing smokers and chewers, where the continued use of pituri is possible within a hospital, as opposed to the need for a smoker to leave the hospital grounds for a cigarette.

The research conveniently enrolled participants who were considered to be over 28 weeks gestation based on the expected due date. Mothers who experienced early- and mid-pregnancy adverse outcomes or birth or who were transferred to a tertiary health service prior to that time were therefore not invited to participate and this may underestimate the impact of nicotine exposure on pregnancy outcomes.

Although 73 mothers and their babies were recruited, it was not possible to obtain all sample types from everyone and the small numbers for each biological sample limits the ability to perform statistical comparisons or draw generalised conclusions. Nevertheless, these results have been reported to inform future studies. Missing data may have biased the results but sensitivity analyses were not conducted for this report. Analytes with concentrations below the level of detection were mathematically added. Furthermore, there are a variety of Australian *Nicotiana* spp. plants which grow across the expanse of Australia with the nicotine content of the individual plants impacted by their location and environmental factors such as soil, rain and temperature and their preparation for use as SLT [6, 9]. Therefore, extrapolation of the findings from this research with other Australian Aboriginal and Torres Strait Islander populations, and other Indigenous worldwide groups and non-Indigenous groups, may not be appropriate.

## Conclusion

The most significant, individually modifiable factor associated with improved maternal and neonatal outcomes is the absence of maternal tobacco smoking. For Central Australian Aboriginal maternal populations, tobacco exposure includes the chewing of wild tobacco plants. Pituri chewing is embedded in Central Australian communities, existing as an early life ‘rite of passage’ from parent to child [9]. The pituri chewer participants may have been exposed to nicotine from the time of their conception via their own mother’s chewing, with continued exposure through breast feeding, followed by their uptake of pituri use in very early life. Here our analysis of NM concentrations from various biological samples provided by mothers who chewed pituri indicates there is the potential for a relationship between nicotine exposure from chewing these plants during pregnancy and adverse maternal and neonatal outcomes, and that these outcomes may be similar to the use of smoked tobacco. However, pituri is not recognised as a tobacco plant by many Aboriginal people or health professionals. Developing health education materials for both populations will provide a significant platform to engage in meaningful tobacco-use discussions, assessments and provide a pathway for cessation strategies.

Importantly, this research demonstrated that cohort mean birthweight (as a sentinel neonatal outcome variable) can be a misleading measure if not considered alongside the presence of elevated maternal glucose and tobacco use. Consideration of the role that nicotine exposure has in the development of elevated maternal glucose in pregnancy is important in mitigating the development of chronic health illness in later life. The long-term effects of the dual exposure to nicotine and elevated glucose on the developing foetus are important to public health knowledge and public health education. Future research that explores the pharmacodynamics and pharmacokinetics of nicotine metabolism in this population will provide important understandings related to these outcomes but is reliant on the collection of accurate tobacco and nicotine exposure data. Modifying the pregnancy health assessment and data collection tools to record the broader range and use of nicotine-containing products will enable a more inclusive and discriminative assessment of their effects on contemporary Australian pregnancies and outcomes.

## Supplementary Information

**Additional file 1:**

## Data Availability

The data generated or analysed during this aspect of the study is included in this published article.

## Abbreviations

AC: Arterial cord blood
AF: Amniotic fluid
AHW: Aboriginal Health Worker
ALO: Aboriginal Liaison Officer
BM: Breast milk
CI: Confidence interval
CS: Caesarean section
g: Gram
LGA: Large for gestational age
LBW: Low birthweight
HBW: High birthweight
IUGR: Intrauterine growth retardation
MB: Maternal venous blood
mL: Millilitres
MU: Maternal urine
nAChRs: Nicotinic acetylcholine receptors
NT: Northern Territory
NU1: Neonatal urine Day 1
NU3: Neonatal urine Day 3
OR: Odds ratio
SCN: Special Care Nursery
SD: Standard deviation
SGA: Small for gestational age
SIDS: Sudden infant death syndrome
SUDI: Sudden unexpected death in infancy
SLT: Smokeless tobacco
NM: Nicotine and metabolites
VC: Venous cord blood
WHO: World Health Organization
