# Supplementary material for: Higher blood nicotine concentrations following smokeless tobacco (pituri) and cigarette use linked to adverse pregnancy outcomes for Central Australian Aboriginal pregnancies
Source: BMC Public Health. 2022 Nov 23;22:2157. doi: 10.1186/s12889-022-14609-4 (PMC9685874; doi:10.1186/s12889-022-14609-4)
Supplement: Supplementary file 1 — Additional file 1: [file 12889_2022_14609_MOESM1_ESM.docx]

**Additional file 1**

**Biochemical Sample Analysis Methods**

**Biochemical Materials and Methods for Biological Sample Analysis**

**1.1. Chemicals**

The following alkaloids and deuterated internal standards were obtained from Toronto Research Chemicals (Toronto, ON, Canada): (R,S)-Anabasine (AB) and (R,S)-Anabasine-2,4,5,6-d4 (AB-d4); (R,S)-Anatabine (AT) and (R,S)-Anatabine-2,4,5,6-d4 (AT-d4); (-)-Cotinine (COT); (+/-)-Nornicotine (NNIC); nicotine-N-β-glucuronide (NIC GLUC); trans-3’-hydroxycotinine (3HC). (-)-Nicotine (NIC), (+/-)-Nicotine-d4 (NIC-d4), (+/-)-Cotinine-d3 (COT-d3) and (+/-)-Nornicotine-d4 (NNIC-d4) were purchased from Sigma (St. Louis, MO, USA). Nicotine-N-β-glucuronide-methyl-d3 (NIC GLUC-d3), trans-3’-hydroxycotinine-d3 (3HC-d3), (1'S, 2'S)-Nicotine 1'-Oxide (NNO) and (+/-) trans-Nicotine-1'-oxide-methyl-d3 (NNO-d3) were obtained from Santa Cruz Biotechnology (Santa Cruz, CA, USA). The purity of all the commercially available reference compounds was ≥98%. LC–MS grade formic acid (>99%) and ammonium formate (>99%) were purchased from Fisher Chemical (Geel, Belgium) and Agilent Technologies (Palo Alto, CA, USA), respectively. Methanol and acetonitrile were purchased from Merck (Darmstadt, Germany) and ammonium hydroxide from Sigma. Ultra-pure water was prepared by a Millipore Milli-Q system (Milford, MA, USA). Stock solution for the alkaloid standards was prepared in methanol at a concentration of 1 mg/mL and was stored at -80°C. Working solutions were prepared by serial dilution from the stock solutions and stored at -20°C until required for an analytical run. Internal standard mixture was prepared by addition of the eight-deuterated internal standards at 1 µg/mL concentration in methanol and stored at -20°C.

**1.2. Sample preparation**

The native fluids (urine, serum, amniotic fluid and breast milk) were thawed and centrifuged at 12,000 x g for 5 min to remove solid impurities.

***1.2.1. Urine***

Urine (20 µL) was spiked with 5 µl of 1 µg/mL internal standard mixture and basified with 1 mL of 0.15 M aqueous-ammonium hydroxide solution. Following brief vortex, the solution was loaded onto the conditioned (1 mL methanol) and equilibrated (1 mL 0.15 M ammonium hydroxide) SampliQ Polymer SCX (30 mg, 1 mL, Agilent Technologies). The loaded cartridge was then washed with 1 mL of 2% formic acid and eluted with 2 mL of methanol and subsequently 2 mL of 3% ammonium hydroxide in methanol. The eluent collected in a 16 x 100 mm glass tube was evaporated in a Pierce Reacti-Therm III heating module (Rockford, IL, USA) to approximately 5-10 µL residual volume to avoid loss of volatile compounds such as cotinine and 3-OH cotinine. Subsequently, the residue was reconstituted in 100 µL of methanol and re-centrifuged in a 1.5 mL tube for 5 min at 12,000 rpm and 1 µL of the supernatant was injected into the LC-MS/MS system.

***1.2.2. Serum, breast milk and amniotic fluid***

An aliquot (50 µL) of plasma, breast milk or amniotic fluid was spiked with 5 µL of 1 µg/mL internal standard mixture and then 100 µL of acetonitrile was added to the solution to aid in matrix clean-up. Following vortex-mixing and 10 min of 12,000 rpm centrifugation, the supernatant was transferred to a 16 x 100 mm glass tube and evaporated by nitrogen gas flow at 40°C for 10 min in a Pierce Reacti Therm III heating module. The residue was then reconstituted in 100 µL of methanol, re-centrifuged in 1.5 mL tube for 5 min at 12,000 rpm and 1 µL of the solution was injected into LC-MS/MS system.

**1.3. LC-MS/MS analysis**

Liquid chromatographic separation was achieved on a Poroshell 120 HILIC column (2.1 x 150 mm, 2.7 µm) with a 1290 Infinity inline filter (0.3 µm, Agilent, Santa Clara, CA, USA) with a gradient system consisting of 10 mM ammonium formate with 0.1% formic acid (pH 3.2, A) and methanol with 0.1% formic acid (pH 3.2, B) at a flow rate of 200 µL/min. The initial mobile phase condition was 5% A which increased linearly to 30% for 3 min followed by an increase to 40% until 5 min, then decreased back to the initial mobile phase condition of 5% A within 1 min and re-equilibrated until 30 min.

Chromatographic analysis for urine and plasma was carried out using a hybrid triple quadruple mass spectrometer API 3000 equipped with a turbo ion spray source, operating in positive ESI mode supported by Analyst software (AB Sciex, Darmstadt, Germany). For the MS/MS scan, the precursor ions were selected, and the products ions were generated using different collision energy; the most intense product ion was selected for the multiple reaction monitoring mode (MRM) scan (Table S1). All MRM transitions were monitored with unit mass resolution for both precursor and product ions. The turbo ion spray source was maintained using the flowing setting: ion spray voltage = 5000 V, turboprobe temperature = 300°C, heater gas flow 7 L/min, nebuliser setting (GAS1) = 15.

Chromatographic analysis for breast milk and amniotic fluid was conducted using an Agilent 6460 Triple Quadrupole tandem mass spectrometer equipped with a Jet Stream source as the detector with Mass Hunter Workstation software (Agilent Technologies, Santa Clara, CA, USA). The mass spectrometer was operated in electrospray positive mode using MRM data acquisition that optimised by highest possible sensitivity for each alkaloid (Table S2). The following ESI conditions were applied: gas flow = 5 L/min, sheath gas flow = 12 L/min, gas temperature = 300°C and sheath gas temperature = 250°C.

**1.4. Method validation**

The linearity of the method was assessed using six serial dilutions from the alkaloid stock solution and internal standard by linear regression analysis. Each dilution was used to construct the calibration plots by plotting the peak area ratio of each alkaloid and its deuterated internal standard *versus* the concentration of alkaloid. The limits of detection (LOD) and limits of quantitation (LOQ) for each alkaloid were determined based (Table S3) on the concentrations (as peak heights) corresponding to 3 x noise and 10 x noise respectively. Precision was assessed using 10 individual aliquots of the fluid samples followed by the calculation of relative standard deviation (RSD) for the peak area ratio of each alkaloid and its deuterated internal standard. Recovery was calculated as the measured concentrations divided by the expected concentrations by using six aliquots each of spiked and non-spiked fluid samples. Each aliquot of milk and serum was spiked to give an added concentration similar to the mid-point of the calibration range.

Table S1. MRM parameters for analysis of alkaloids in urine and serum

|  | Analyte | Precursor ion (m/z) | Product ion  (m/z) | Collision energy (V) |
| --- | --- | --- | --- | --- |
| 1 | AB  AB-d4 | 163.0  167.0 | 92.0  96.0 | 29  29 |
| 2 | AT  AT-d4 | 160.9  164.9 | 144.1  148.1 | 17  17 |
| 3 | COT  COT-d3 | 177.0  180.0 | 80.1  80.1 | 35  35 |
| 4 | NNIC  NNIC-d4 | 149.1  153.1 | 132.0  132.0 | 17  17 |
| 5 | NIC GLUC  NIC GLUC-d3 | 339.1  342.1 | 163.4  166.4 | 21  21 |
| 6 | 3HC  3HC-d3 | 193.1  196.1 | 80.2  80.2 | 37  37 |
| 7 | NNO  NNO-d3 | 179.0  182.0 | 132.1  132.1 | 23  23 |
| 8 | NIC  NIC-d4 | 163.1  167.2 | 130.0  134.2 | 23  23 |

Legend: Multiple reaction monitoring mode (MRM) scan - Urine and Serum

Table S2. MRM parameters for analysis of alkaloids in breast milk and amniotic fluid

|  | Analyte | Precursor ion  (m/z) | Production  (m/z) | Collision energy  (V) |
| --- | --- | --- | --- | --- |
| 1 | AB  AB-d4 | 163.1  167.1 | 80.1  84.0 | 24  24 |
| 2 | AT  AT-d4 | 161.1  165.1 | 144.0  148.0 | 12  12 |
| 3 | COT  COT-d3 | 177.1  180.1 | 80.1  80.1 | 28  28 |
| 4 | NNIC  NNIC-d4 | 149.1  153.1 | 80.1  80.0 | 24  24 |
| 5 | NIC GLUC  NIC GLUC-d3 | 339.2  342.2 | 163.1  166.1 | 12  12 |
| 6 | 3HC  3HC-d3 | 193.1  196.1 | 80.1  80.1 | 32  32 |
| 7 | NNO  NNO-d3 | 179.1  182.1 | 132.1  132.1 | 16  16 |
| 8 | NIC  NIC-d4 | 163.1  167.1 | 130.0  134.0 | 24  24 |

Legend: Multiple reaction monitoring mode (MRM) scan – Breast milk and amniotic fluid

Table S3. Limit of Detection (LOD) and Limit of Quantification (LOQ) for analysis of alkaloids

| Analyte | LOD (ng/mL) | LOQ (ng/mL) |
| --- | --- | --- |
| AB | 0.074 | 0.223 |
| AT | 0.073 | 0.222 |
| COT | 0.410 | 1.241 |
| NNIC | 1.215 | 3.682 |
| NIC GLUC | 2.146 | 6.502 |
| 3HC | 1.045 | 3.167 |
| NNO | 0.255 | 0.772 |
| NIC | 0.888 | 2.690 |
